# Biobanking after robotic-assisted radical prostatectomy: a quality assessment of providing prostate tissue for RNA studies

**DOI:** 10.1186/1479-5876-9-121

**Published:** 2011-07-26

**Authors:** Harveer Dev, David Rickman, Prasanna Sooriakumaran, Abhishek Srivastava, Sonal Grover, Robert Leung, Robert Kim, Naoki Kitabayashi, Raquel Esqueva, Kyung Park, Jessica Padilla, Mark Rubin, Ashutosh Tewari

**Affiliations:** 1 Lefrak Center of Robotic Surgery & Institute for Prostate Cancer, Brady Foundation Department of Urology, Weill Cornell Medical College, New York, NY, USA; 2 Department of Pathology and Laboratory Medicine, Weill Cornell Medical College, New York, New York, USA

**Keywords:** biobanking, prostate collection, ischaemia time, robotic-assisted radical prostatectomy, RNA quality, RIN

## Abstract

**Background:**

RNA quality is believed to decrease with ischaemia time, and therefore open radical prostatectomy has been advantageous in allowing the retrieval of the prostate immediately after its devascularization. In contrast, robotic-assisted laparoscopic radical prostatectomies (RALP) require the completion of several operative steps before the devascularized prostate can be extirpated, casting doubt on the validity of this technique as a source for obtaining prostatic tissue. We seek to establish the integrity of our biobanking process by measuring the RNA quality of specimens derived from robotic-assisted laparoscopic radical prostatectomy.

**Methods:**

We describe our biobanking process and report the RNA quality of prostate specimens using advanced electrophoretic techniques (RNA Integrity Numbers, RIN). Using multivariate regression analysis we consider the impact of various clinicopathological correlates on RNA integrity.

**Results:**

Our biobanking process has been used to acquire 1709 prostates, and allows us to retain approximately 40% of the prostate specimen, without compromising the histopathological evaluation of patients. We collected 186 samples from 142 biobanked prostates, and demonstrated a mean RIN of 7.25 (standard deviation 1.64) in 139 non-stromal samples, 73% of which had a RIN ≥ 7. Multivariate regression analysis revealed cell type - stromal/epithelial and benign/malignant - and prostate volume to be significant predictors of RIN, with unstandardized coefficients of 0.867(p = 0.001), 1.738(p < 0.001) and -0.690(p = 0.009) respectively. A mean warm ischaemia time of 120 min (standard deviation 30 min) was recorded, but multivariate regression analysis did not demonstrate a relationship with RIN within the timeframe of the RALP procedure.

**Conclusions:**

We demonstrate the robustness of our protocol - representing the concerted efforts of dedicated urology and pathology departments - in generating RNA of sufficient concentration and quality, without compromising the histopathological evaluation and diagnosis of patients. The ischaemia time associated with our prostatectomy technique using a robotic platform does not negatively impact on biobanking for RNA studies.

## Introduction

Prostate cancer remains the most common non-dermatological malignancy in men in the Western world [1]. As our knowledge of prostate cancer continues to be driven by genomic studies, the accumulation of high quality tissue within established biobanks becomes increasingly important. High-throughput cDNA microarrays are being used to map gene expression profiles in prostate tissue, leading the way for improved disease classifications, prognostic indicators, and therapeutic targets via a greater understanding of the pathogenesis of prostate cancer [2]. In order to draw meaningful conclusions from these transcriptomes, investigators must possess robust methods for tissue biobanking, as well as regularly perform quality control on the samples they collect. Variations in both biobanking protocols and quality control methods can limit comparisons between different research groups, and hence the veracity of any conclusions drawn from their molecular profiles.

The last ten years has witnessed significant advances in radical prostatectomy, with the incorporation of robotic platforms into the procedure. Robotic-assisted laparoscopic radical prostatectomy (RALP) has become the most widespread treatment for organ-confined prostate cancer, currently accounting for more than 75% of all radical prostatectomies performed in the USA [3]. The technique aims to minimize patient morbidity and improve convalescence while delivering high standards of oncological and functional control [4]. One inevitable consequence of this transition has been the impact of RALP on specimen collection. Once the prostate has been freed from all its anatomical attachments, it remains within the body until later steps of the operation (including the vesico-urethral anastomosis) have been completed. Concern has surrounded the impact of warm ischaemia on the integrity of prostate samples that are subsequently banked and used for genetic analysis. Few studies have reported the RNA quality of prostate cancer samples derived from RALP, and of these, small sample sizes of specimens may potentially limit their reproducibility [5,6].

Different methods of assessing RNA quality have further complicated efforts to ensure consistency between biobanks. Spectroscopic techniques compare the absorbance of 260 nm and 280 nm ultraviolet light by nucleic acids and proteins respectively. This so-called ratios method of assessing RNA quantity and purity has been shown to be ambiguous when compared to subjective expert evaluations of microcapillary electrophoretic traces [7,8]. In order to standardize the process of interpreting RNA quality, Agilent Technologies (Santa Clara, CA) have developed the RNA Integrity Number (RIN) - a software algorithm which allows for the classification of total RNA, based on a numbering system from 1 (most degraded) to 10 (intact) [9]. Using the Agilent 2100 Bioanalyser and lab-on-chip microfluids technology, software is able to generate an electropherogram; the RIN algorithm then generates its integrity number by taking into account the entire trace. This removes any user-dependence which can often limit manual methods, and hence allows the direct comparison of specimen RNA quality between different institutions. The advantages of RIN over other analytical methods have been supported by several groups [8,10], and it has subsequently become widely employed in studies which seek to establish RNA quality [6,11-13].

In addition to the effect of warm ischaemia, other clinicopathological correlates of RNA quality may be considered. Prostates have been shown to be exquisitely sensitive to intraoperative manipulation, showing changes in gene expression well before devascularization of the prostate [14,15]. It has also been suggested that the histological properties of a sample, and its location within a specimen, may influence the quality of RNA obtained [11].

In this paper, we report the methodology of tissue collection in our RALP prostate cancer biobank involving 1709 radical prostatectomy specimens. We validate the robotic prostatectomy procedure as a reliable source for prostate cancer tissue collection, using RIN values from more than 140 specimens, and consider the effects of various clinicopathological variables on specimen quality.

## Materials and methods

### Ethical approval and patient consent

An Institutional Review Board-approved research protocol was obtained in November 2006 for the collection of prostate samples after robotic prostatectomy for the treatment of clinically localized prostate cancer. Consent was obtained from each patient prior to them entering surgery, following a detailed review of the patient consent form.

### Tissue collection

In order to ensure consistency, all prostates within the RALP prostate cancer biobank were derived entirely from our institution, led by a single surgeon (AT), and using our previously reported technique of RALP [4]. The prostates were extirpated within an EndoCatch bag, before being assessed by the console surgeon or trained assistants. The specimens were then transported by the robotic team to the pathology department without delay (and hence overcoming the need for temporary ice storage), where they were received by a technician for immediate preparation.

### Specimen preparation

**Figure 1:**
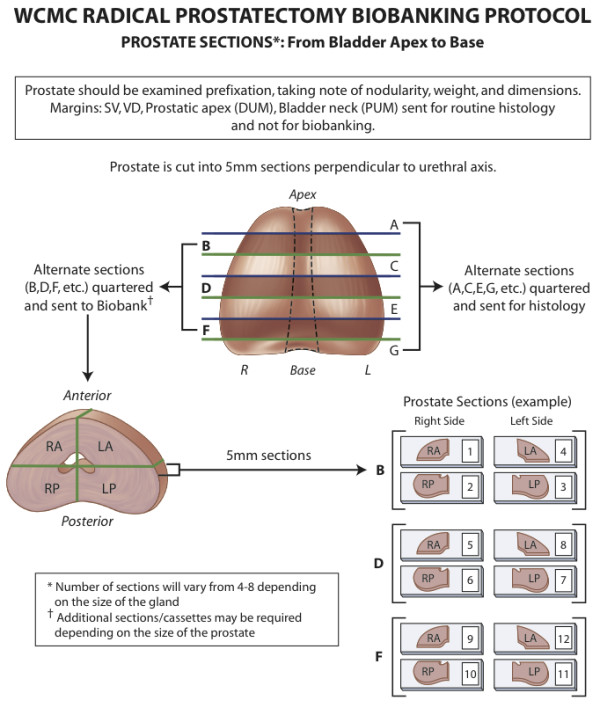
**WCMC radical prostatectomy biobanking protocol**.

**Figure 2:**
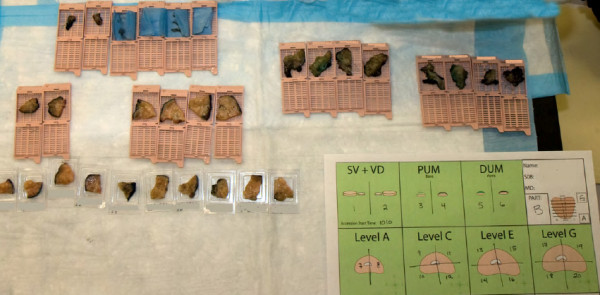
**A photograph showing the labelled cassettes of prostate tissue from a single prostate specimen prior to snap freezing**.

The prostate was weighed, orientated, and marked in black and green ink for the left and right sides respectively. Margin analysis was initially performed from tissue cassettes containing seminal vesicles and vasa deferentia, the apex (distal urethral margin), and the bladder neck (proximal urethral margin). Serial sections of the prostate, perpendicular to the urethra and measuring 5 mm in thickness, were then taken from the bladder base to the apex, and alphabetically labeled (e.g. A to H). Each section was subsequently quartered or divided into six equal parts (depending on the prostate size), for placement into individual cassettes. Alternate sections (e.g. A, C, E and G) and 'margin' samples were then formalin-fixed for routine histopathological diagnosis by immersing the tissue in 10% neutral buffered formalin for between 4 and 24 hours, before being processed and embedded in paraffin. The remaining alternate prostate sections (i.e. B, D, F and H) were then coated in Optimal Cutting Temperature (OCT) media (Sakura Finetek, Torrance, CA), prior to snap freezing in liquid nitrogen and storage in a plastic specimen bag at -80°C in our tissue laboratory. The process of specimen collection is illustrated in Figure 1 and a photograph exemplifying the samples collected from a prostate specimen is presented in Figure 2.

### Histological characterization of banked specimens

Following the establishment of the RALP prostate cancer biobank in February 2007, a Haematoxylin & Eosin stained microscopic slide was prospectively prepared for each banked tissue sample, prior to snap freezing of the prostate sections. The percentage of epithelial cells present within the tumour foci was determined by an expert histopathologist (RE), and a cut-off of 90% was used as a determinant of either benign, tumour or stromal classification. The histopathologist then demarcated the areas of tumour, benign, and stromal tissue on each slide. From October 2007 onwards, as a result of our biobanking protocol being approved by our IRB, samples were continuously collected and included on the grounds of fulfilling the criterion of having foci of more than > 90% pure cell populations. From these 142 specimens 186 samples were derived. In order to ensure that these samples of specimens with pure cell populations > 90% were representative of the population, further statistical analysis confirmed that there were no significant differences between the study population of 142 specimens and the entire biobanked population of 1709 (see Table 1).

**Table 1:** Preoperative variables, baseline demographics and operative data of 142 specimens, and comparison with remainder population.

Variable	**n**^**1**^	Mean sample group (SD)	Mean remainder group (SD)	p-value
Age	142	61.4 years (6.7)	60.3 years (7.1)	0.230
Body Mass Index	125	27.3 kg/m^2^(3.7)	27.0 kg/m^2^(3.9)	0.778
Preoperative PSA level	141	6.5 ng/ml (3.8)	5.9 ng/ml (5)	0.157
Total operating time	126	142 min (33)	151 min (37)	0.445
Estimated blood loss	137	157 ml(32)	162 ml(30)	0.546

^1^Missing data reflects incomplete data collection.

Due to the multifocal and heterogeneous nature of prostate cancer tissue, samples derived from the same specimen were considered to be independent. A matched pair analysis between stromal and benign epithelial samples taken from the same specimen was performed (see Table 2).

**Table 2:** Results from matched pair analysis between 45 stromal and 45 epithelial samples taken from the same specimen.

Sample	Mean	SE	CI	p-value
Epithelial	6.496	0.217	-	-
Stromal	4.907	0.238	-	-
Epithelial-Stromal	1.589	0.234	1.118/2.060	< 0.001

### RNA quality assessment

The corresponding 5 mm frozen tissue blocks for each of these 142 banked samples were aligned with their appropriate slides. The demarcated cell type area (tumour, benign or stromal) was identified and once the tissue block had been sufficiently thawed (while remaining at sub-zero temperatures) two to three 1.5 mm cores were taken using biopsy punches (Miltex, York, PA) for RNA extraction, which was performed using an Invitrogen (Carlsbad, CA) protocol. Briefly, the tissue core was homogenized in 1 ml of TRIzol (Invitrogen) and left at room temperature for 5 min; 200 μl of chloroform was added, and phase separation was achieved by centrifugation (12 000 g, 15 min, 4°C). Next, 10 μg glycogen and 500 μl isopropanol were added to the aqueous RNA-containing phase and incubated for 5 min at room temperature, in order to precipitate the RNA (12 000 g, 10 min, 4°C). The supernatant was then carefully removed, before the addition of 1 ml 75% ethanol, and further centrifugation (7 500 g, 10 min, 4°C). The remaining ethanol was removed by air-drying for 5-10 min, before dissolving the precipitate in 20-30 μl of RNase free water. The samples were finally treated with a DNA-free Kit (Ambion, Austin, TX) according to the manufacturer's instructions.

RNA concentration was measured using NanoDrop 1000 or NanoDrop 8000 spectrophotometers (Thermo Scientific, Waltham, MA). The RIN numbers for the RNA samples were then measured using the Agilent 2100 Bioanalyzer (Santa Clara, CA) with RNA 6000 Nano Labchip kit according to the manufacturer's instructions.

### RALP database

Pre, intra- and postoperative clinical data was prospectively collected in our RALP database. This included patient age, body mass index, preoperative prostate specific antigen, total operative time, estimated blood loss, prostate volume, the presence of any positive surgical margin, Gleason score, percentage cancer, pathological stage and storage time. For 49/142 (34.5%) samples, the total warm ischaemia time (total WIT) was measured along with the time from prostate devascularisation to extirpation (intraoperative time), sample collection (collection time), and pathology processing (time until pathology specimen is flash frozen at -80°C). Ischaemia time was shown to be relatively constant, and hence was only recorded for 49 samples.

### Statistical analysis

Clinicopathological variables for 186 samples were evaluated for correlation with the dependent variable - RIN - using multiple linear regression. The following independent variables were inputted and backward Wald selection was used to identify the best model: stromal/epithelial cells, benign/malignant cells, patient age, body mass index, preoperative prostate specific antigen, prostate volume (< 40 g and ≥ 40 g), Gleason score (< 7 and ≥ 7), percentage cancer, pathological stage (< pT3 and ≥ pT3), presence of positive surgical margin, estimated blood loss, total WIT (including intraoperative time, collection time and processing time), total operating time, and storage time (number of months between flash freezing and RNA extraction). Subgroup analysis was performed using the student t-test for the statistically significant variables included in the best model. All statistical analysis was performed using SPSS (v18.0 for Windows; IBM, Armonk, NY).

## Results

Between January 2007 and August 2010, 1709 prostate specimens were consistently collected and stored in our RALP prostate cancer biobank (see Table 3). The baseline demographics and preoperative variables of the 142 prostate specimens used for this study are shown in Table 1. Mean total operating time and estimated blood loss for the 142 patients was 142 min and 157 ml respectively. The mean prostate volume was 51 ml. A summary of the pathological and specimen variables for this cohort is shown in Table 4.

**Table 3:** Number of specimens collected and stored in the RALP prostate cancer biobank.

Year	Number of specimens
2007^1^	437
2008	440
2009	524
2010	308^2^

^1^RIN values in this study were derived from samples prepared since October 2007 after the introduction of the technique by one of the authors (MR); ^2^As of August 2010

**Table 4:** Pathological and specimen data.

Variable	n	Mean or % (SD)
Intraoperative time	49	43 min (18)
Collection time	49	31 min (17)
Processing time	49	45 min (16)
Total WIT	49	120 min (30)
		
Prostate volume	137	51 ml (28)
		
Gleason sum		
< 7	21	15%
7	105	77%
> 7	11	8%
		
Positive margin rate %	22	16%
		
Pathological stage		
T2	97	72%
≥ T3	38	28%
		
Storage time, months	52	8.7 months (7.3)
		
RNA concentration	142	692 ng/μl(441)
Sample RIN		
Stromal	47	4.91 (1.67)
Epithelial	139	7.25 (1.64)
Benign	112	5.98 (1.91)
Malignant	74	7.70 (1.45)

Between 2 and 3 sections of the biobanked specimens were retained, equating to 8 to 12 frozen tissue blocks or approximately 40% of the total prostate body per specimen (see Figure 2).

**Figure 3:**
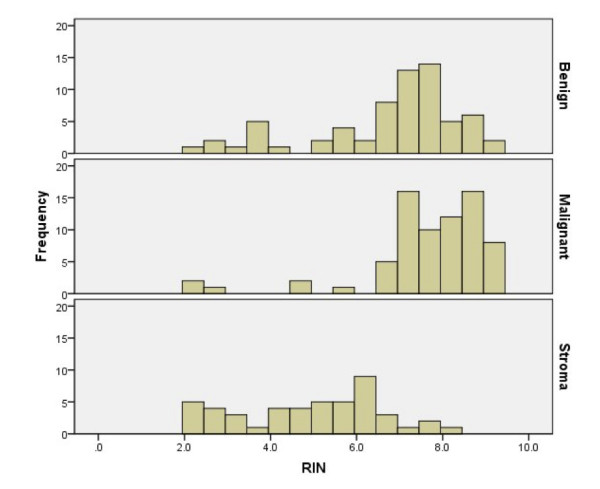
**Histograms to show the distribution of RINs for benign, malignant and stromal samples**.

RNA was isolated from 186 samples and analyzed using the Agilent Bioanalyzer 2100. Two stromal samples were excluded from analysis for failing to generate any RIN values, likely as a result of DNA or RNAse contamination. The mean concentration of RNA obtained was 692 ng/μl (standard deviation 441 ng/μl). The histograms of RINs obtained for benign, malignant and stromal specimens are presented in Figure 3.

A mean RIN 4.91 (n = 47 s.d.1.67) for stromal and 7.25 for epithelial (n = 139, s.d.1.64) was found, which reached statistical significance (p < 0.001). Hence we were able to demonstrate a mean RIN of 7.25 in 139 non-stromal samples, 73% of which had a RIN ≥ 7.

112 benign and 74 tumour samples were identified, with significantly different mean RINs of 5.98 (s.d.1.91) and 7.70 (s.d. 1.45) respectively. Within the epithelial cohort, benign and tumour samples demonstrated mean RINs of 6.76 (s.d.1.45, n = 66) and 7.70 (s.d.1.46, n = 73) respectively (p < 0.001).

Multivariate regression analysis was performed, and cell type - stromal/epithelial and benign/malignant - and prostate volume were found to be significant predictors of RIN, with unstandardized coefficients of 0.867 (p = 0.001), 1.738 (p < 0.001) and -0.690 (p = 0.009) respectively.

There was also a significant trend between total operating time and RIN (B = -0.012, p = 0.050). Spearman's rank correlation between total operating time and prostate volume verified a significant negative correlation (ρ = -0.210 p < 0.001).

There were no other clinicopathological variables that were found to be statistically significant (p < 0.05) predictors of RIN (see Table 5). Subgroup analysis using the student t-test, revealed a mean RIN of 7.5 and 6.3 for prostates < 40 g (n = 135) and ≥ 40 (n = 51) respectively (p < 0.001).

**Table 5:** Univariate and multivariate linear regression analysis between various clinicopathological variables and RIN for 186 prostate samples^1^.

	Univariate				Multivariate^2^			
Variable	B	SE	CI (95%)	p-value	B	SE	CI (95%)	p-value
Constant	-	-	-	-	6.395	0.531	5.347/7.444	< 0.001
Benign/malignant^3^	1.724	0.261	1.209/2.238	< 0.001	0.867	0.261	0.352/1.382	0.001
Stromal/epithelial^4^	2.336	0.278	1.789/2.884	< 0.001	1.738	0.292	1.161/2.316	< 0.001
Age	-0.011	0.021	-0.054/0.031	0.600	-	-	-	-
Body Mass Index	-0.048	0.042	-0.131/0.035	0.256	-	-	-	-
Preoperative PSA	-0.015	0.039	-0.091/0.062	0.709	-	-	-	-
Prostate volume	-1.181	0.306	-1.784/-0.577	< 0.001	-0.690	0.262	-1.207/-0.173	0.009
Gleason sum	0.093	0.409	-0.715/0.900	0.821	-	-	-	-
% cancer	0.002	0.021	-0.040/0.043	0.939	-	-	-	-
Pathological stage	0.254	0.336	-0.410/0.917	0.451	-	-	-	-
Positive surgical margin	0.645	0.446	-0.234/1.525	0.150	-	-	-	-
Estimated blood loss	-0.002	0.005	-0.011/0.008	0.735	-	-	-	-
Intraoperative time	-0.017	0.011	-0.039/0.005	0.136	-	-	-	-
Collection time	-0.001	0.12	-0.024/0.023	0.956	-	-	-	-
Processing time	-0.012	0.012	-0.036/0.012	0.313	-	-	-	-
Total WIT	-0.010	0.007	-0.022/0.003	0.147	-	-	-	-
Total operating time	-0.012	0.004	-0.019/-0.004	0.003	-0.006	0.003	-0.012/0.000	0.050
Storage time	0.067	0.067	-0.066/0.200	0.323	-	-	-	-

^1^All 186 samples were included in the regression analysis with the *n *number of each variable corresponding to those in Tables 1 and 3; ^2^Multivariate linear analysis using the best model; ^3^Benign (0), malignant (1); ^4^stromal (0), epithelial (1).

Abbreviations: B, unstandardised correlation coefficient; CI, 95% confidence interval; SE, standard error; WIT, warm ischaemia time.

## Discussion

We report a reliable method of tissue banking which does not compromise the histological evaluation of prostate samples for patient diagnosis. By flash freezing tissue sections from the prostate, conventional histological evaluation can be performed without compromising margin analysis or pathological staging. In the rare event that an area of suspicion is only identified at the border of a prostate section, the adjacent biobanked section can be retrieved from storage for further study by the pathologist. 9% of our patients have more tissue taken from the biobank after identifying suspicious areas on clinical specimens, and we believe that the ability to access the biobanked tissue is fundamental to ensuring the integrity of the histological diagnosis.

Harvesting alternate sections also ensures the procurement of a substantial mass of tissue, and therefore provides a sufficient yield of RNA for genetic studies. Furthermore, the tissue is of sufficient quality for use in high-demand genetic studies, with 73% of epithelial samples demonstrating a RIN > 7. However, the converse is equally true, and we should note that 27% of epithelial samples will be insufficient for high fidelity RNA studies.

A RIN of > 7 is generally considered suitable for gene expression studies [13], and while our study did not have a control arm, the user-independence of measuring RIN values permits the comparison between different studies and helps to overcome this limitation. A large report from a cooperative human tissue biobank demonstrated 'less than good' quality RNA in 40% of samples collected [16], while a large pancreatic cancer biobank has demonstrated RIN ≥ 7 in just 42% of samples [13]. While it is reasonable to assume that some of these differences reflect the varying cellular content of different tissues (pancreas being more sensitive to degradation than prostate), it is also possible that the delicate tissue-handling capabilities afforded by the robotic platform are responsible for a less severe impact on the cellular response to surgery; a possible relationship between RNase release within the tissue and specimen handling intraoperatively has been suggested [11]. It must be reiterated that a direct comparison was not performed, and obviously the ideal randomized controlled comparison study to elucidate any difference would be unethical. To date, any comparisons between less mature robotic series and traditional open radical procedures have failed to show any significant difference in RNA quality [5,6]. Comparing our results with data from other groups is challenging, and in part is limited by the small sample sizes, with Ricciardelli et al. reporting RIN values of 8-10 from just five prostate specimens [6]. Bertilsson and colleagues have since reported RIN scores above 9 using further modified techniques with 53 prostate samples, presumably using the same source of samples from open radical prostatectomy [17]. Such differences even between studies from the same institution highlight the important principle that RNA integrity reflects a complex interplay between pre-processing collection methods, and tissue processing methodology. From our data in the context of limited external data, we surmise that RALP permits the collection of prostate specimens which are at least non-inferior to traditional open prostatectomy with respect to RNA integrity.

The two stromal samples excluded from our multivariate analysis reflects the sensitivity of the RIN protocol to local DNA and/or RNase contamination. The method we have described is particularly advantageous in permitting the histological identification of our banked specimens, in comparison to biopsy techniques which rely on less accurate methods of sampling [18]. For example, Riddick et al. have described taking punch biopsies from suspicious areas of the prostate (as identified by examining the prostate for firm irregular nodules and/or colour/texture heterogeneity), and performing histopathological assessment of the surrounding excised area. Although this method demonstrated concordance with the core sample in 92% of cases, it cannot be used to target specific cell populations within the biobanked tissue. We are able to direct our biopsy cores to histological areas of interest, permitting the investigation of stromal, benign or malignant epithelial cells. Since our samples are 5 mm in diameter there is a potential for introducing alternative cell types to that identified in the corresponding slide, although this method was consistent for all of our studies, thus minimising any bias which may have been introduced; alternative strategies such as Laser Capture Microdissection may offer greater selectivity and improve cell selection, but require real-time pathology support which is not available at most institutions including our own.

While a few samples were derived from the same RALP specimen, due to the multifocality and heterogeneity of malignant prostatic tissue, it is reasonable to assume that such samples will behave independently, hence minimising any selection bias which this might have introduced. Matched pair analysis between 45 stromal and 45 benign epithelial samples taken from the same specimen, confirmed the same relationship between RIN and cell type, with stromal samples showing a significantly lower mean RIN than epithelial samples (see Table 2).

Although it appears reasonable to assume that longer ischaemia times will potentiate RNA degradation, in this study we have found no negative impact on RNA quality within the narrow warm ischaemia times of robotic prostatectomy (mean total WIT of 120 mins). In one time course degradation study of lung tissue, nucleic acid stability has been demonstrated for up to 5 hours after excision at room temperature [16]. Analysis of non-fixed surgical specimens revealed RNA stability in fresh tissue for up to 6-16 hours at room temperature [19]. Similar studies have demonstrated minimal RNA degradation in samples stored on ice for as long as 24-96 hours after collection [12,20]. While gene expression studies suggest an impact of ischaemia on prostatic tissue due to marked changes in hypoxia-related genes within the first hour of surgery [14,15], our results did not show a relationship between warm ischaemia time and RIN (a measure of the integrity of the total RNA population, therefore not discounting alterations in the transciptome). This leads to the suggestion that the onset of cellular ischaemia intraoperatively is sufficient to produce genetic responses, without necessarily compromising the RNA integrity of prostatic tissue. It is possible that the expediency of our surgical technique impairs our ability to extrapolate any relationship with ischaemia, due to our narrow range of operating times. This finding also lends further support to our method of tissue collection.

In an effort to better understand clinicopathological factors which may influence specimen RNA quality, we performed multiple linear regression analysis, and found an inverse relationship between prostate volume and RNA quality.

One explanation for the relationship with prostate volume, may be a greater degree of ischaemia in specimens with a smaller surface area: volume ratio, although this is difficult to rationalize without a relationship between ischaemia time and RIN. Bertilsson et al. described a weak correlation with blood loss (r = -0.11, p = 0.02); the group postulated a relationship between excessive surgical handling, as indicated by blood loss, and subsequent RNase release [11]. We did not anticipate a relationship between blood loss and RNA quality, given the restricted range of this variable (mean 157 ml, standard deviation 41 ml) when using a robotic technique, compared with the open radical prostatectomy study (median 575 ml; > 50% between 500-1000 ml). However, a similar explanation may be used for our relationship with prostate volume, with smaller prostates suffering less intraoperative surgical manipulation, and hence RNase release. An interquartile range was not reported in Bertilsson's initial study, and it is possible that a restricted cohort limited their identification of this correlation. One additional explanation may be that larger prostates are composed of a greater proportion of stromal tissue, which was also shown to inversely correlate with RNA quality in this study (r = -0.34, p = 0.03) [11] as well as our own (B = 1.738, p < 0.001).

The study identified a higher quality of RNA associated with samples taken from tumour cells as opposed to benign cells. It is possible that this relationship is related to a greater abundance of RNA within more aggressive tumour cell populations; this may reflect greater cell turnover and/or a higher rate of transcription per cell. Our hypothesis stems from the tumour cell exhibiting a greater abundance of RNA transcripts, and hence it might be postulated that a greater proportion of intact mRNA may exist, as a function of unregulated synthesis of a limited number of malignant transcripts. However, there is no literature to support this hypothesis and as such it warrants further investigation; we are in the process of designing a future study to evaluate this.

## Conclusions

While not discounting changes in gene expression, we have shown that RALP does not contribute to significant RNA degradation. We have outlined a standardized tissue collection protocol for prostates derived from robotic prostatectomy procedures - representing the concerted efforts of dedicated urology and pathology departments - which ensures consistently high quality of RNA while delivering uncompromised histopathological evaluation.

## Competing interests

The authors declare that they have no competing interests.

## Authors' contributions

The conception and design of the study was by AT and MR. HD, DR, AS, SG, RL, RK, NK, RE, KP and JP were responsible for data acquisition. HD and PS performed the analysis of results and interpretation. The manuscript was drafted and critically revised by HD, PS and AT, and read and approved by all the authors.
